# Evolutionary diversification of *Pseudomonas aeruginosa* in an artificial sputum model

**DOI:** 10.1186/s12866-016-0916-z

**Published:** 2017-01-05

**Authors:** Emily V. Davies, Chloe E. James, Michael A. Brockhurst, Craig Winstanley

**Affiliations:** 1 Institute of Infection and Global Health, University of Liverpool, 8 West Derby Street, Liverpool, L69 7BE UK; 2 School of Environment and Life Sciences, University of Salford, Manchester, M5 4WT UK; 3 Department of Biology, University of York, York, YO10 5DD UK

**Keywords:** *Pseudomonas aeruginosa*, Bacteriophage, Cystic fibrosis, Evolution, Motility, Biofilm, Antimicrobial resistance

## Abstract

**Background:**

During chronic lung infections of cystic fibrosis patients *Pseudomonas aeruginosa* populations undergo extensive evolutionary diversification. However, the selective drivers of this evolutionary process are poorly understood. To test the effects of temperate phages on diversification in *P. aeruginosa* biofilms we experimentally evolved populations of *P. aeruginosa* for approximately 240 generations in artificial sputum medium with or without a community of three temperate phages.

**Results:**

Analysis of end-point populations using a suite of phenotypic tests revealed extensive phenotypic diversification within populations, but no significant differences between the populations evolved with or without phages. The most common phenotypic variant observed was loss of all three types of motility (swimming, swarming and twitching) and resistance to all three phages. Despite the absence of selective pressure, some members of the population evolved antibiotic resistance. The frequency of antibiotic resistant isolates varied according to population and the antibiotic tested. However, resistance to ceftazidime and tazobactam-piperacillin was observed more frequently than resistance to other antibiotics, and was associated with higher prevelence of isolates exhibiting a hypermutable phenotype and increased beta-lactamase production.

**Conclusions:**

We observed considerable within-population phenotypic diversity in *P. aeruginosa* populations evolving in the artificial sputum medium biofilm model. Replicate populations evolved both in the presence and absence of phages converged upon similar sets of phenotypes. The evolved phenotypes, including antimicrobial resistance, were similar to those observed amongst clinical isolates from cystic fibrosis infections.

**Electronic supplementary material:**

The online version of this article (doi:10.1186/s12866-016-0916-z) contains supplementary material, which is available to authorized users.

## Background

*Pseudomonas aeruginosa* is an important opportunistic pathogen that can cause chronic lung infections in patients with cystic fibrosis (CF) [1] or non-CF bronchiectasis [2]. Once established as a chronic infection, a *P. aeruginosa* strain can remain in the lungs of a patient for life, despite frequent use of antibiotic therapy. During the infection, the pathogen adopts a biofilm lifestyle, with evidence from explanted lungs suggesting the formation of free aggregates rather than surface-attached biofilms [3]. Throughout these long time periods, populations of *P. aeruginosa* adapt to the CF lung in several ways, with isolates often exhibiting phenotypes such as mucoidy, auxotrophy, hypermutability, loss of known virulence factors, loss of motility, slower growth in rich media and resistance to antimicrobials [4–7]. In addition to flagella-mediated swimming motility, *P. aeruginosa* exhibits motility on solid or semi-solid surfaces, using IV pili for twitching motility [8], and both flagella and type IV pili for swarming motility [9]. In CF, mutations occur in genes encoding both type IV pili and flagella.

Recently, it has also become evident that *P. aeruginosa* populations in the CF lung can diversify both phenotypically [7, 10–16] and genetically [14, 17]. In particular, we have shown that populations of the most common clone of *P. aeruginosa* infecting CF patients in the UK, the Liverpool Epidemic Strain (LES) [18, 19], are highly diverse [11, 12, 20]. However, the factors driving and maintaining this diversity, also observed in other CF pathogens [21], are poorly understood.

It has been shown that sub-inhibitory concentrations of antibiotics can drive diversification of *P. aeruginosa* populations [22], but other possible contributory factors include host-responses, other members of the complex microbial community associated with the CF lung [23] or the biofilm lifestyle itself [24]. Temperate phages are abundant in the CF lung [25], and we have demonstrated previously that LES temperate phages can both mediate and drive adaptive evolution during experimental evolution in a biofilm model system, by increasing the supply of mutations in genes involved in type IV pilin biogenesis and quorum sensing through insertional inactivation of bacterial genes [26, 27]). The LES phages have also been implicated in the competitiveness of the strain in chronic infection models [28, 29].

To test the hypothesis that temperate phages play an important role in driving phenotypic diversity, here we analysed a suite of phenotypic traits in populations of *P. aeruginosa* that have been experimentally evolved for approximately 240 bacterial generations with or without an assemblage of three LES temperate phages (LESφ2, φ3 and φ4) in Artificial Sputum Medium (ASM). ASM is an in vitro environment that recapitulates the physiochemical properties of lung sputum and in which *P. aeruginosa* forms free-floating biofilms [30] and expresses biofilm-associated genes [31].

## Results

### Phenotypic diversification and loss of wild-type phenotypes occurred in both treatment and control populations

Twelve replicate populations of *P. aeruginosa* PAO1 were experimentally evolved for ~240 generations in ASM in the absence (#1-6) [control treatment] or presence (#7-12) of three temperate LES phages. At the end of the evolution experiment, 40 randomly-selected isolates per population were characterised by assessing a suite of phenotypic traits previously associated with adaptation to the CF lung [7]. These were three types of motility (swimming, swarming and twitching), auxotrophy, hypermutability, and resistance to each of the three LES phages (LESφ2, φ3 and φ4). These data are available in Additional File 1. Extensive phenotypic diversity was observed in all endpoint populations. Analysis of molecular variance (AMOVA) indicated that the majority of the phenotypic variance was associated with diversity within (74%) and between populations (24%), whereas less than 2% of the variation was explained by treatment (i.e. presence/absence of phages) (Table 1). From the 480 isolates, 57 different haplotypes were identified, based on eight phenotypic traits. The haplotype of the ancestor was very rare in the evolved populations; only 8/480 isolates shared the ancestral haplotype, all of which were recovered from the control treatment. The most common haplotype constituted 23% of isolates and was observed in all but one population. This haplotype was characterised by loss of all three types of motility and resistance to all three LES phages, but displayed the wild-type mutation rate and growth on minimal media phenotypes.

**Table 1:** Analysis of molecular variance of the phenotypic diversity of haplotypes identified in endpoint populations

Variance component	σ^2^ Variance	% of total variance (100)	*P*
Within populations	0.773	74	<0.02
Between populations (within treatment)	0.183	24	<0.02
Between treatments	0.015	2	0.26

Variance was partitioned into within and between populations, and between treatments. Significance was tested by Monte-Carlo permutation tests (49 permutations)

**Fig. 1:**
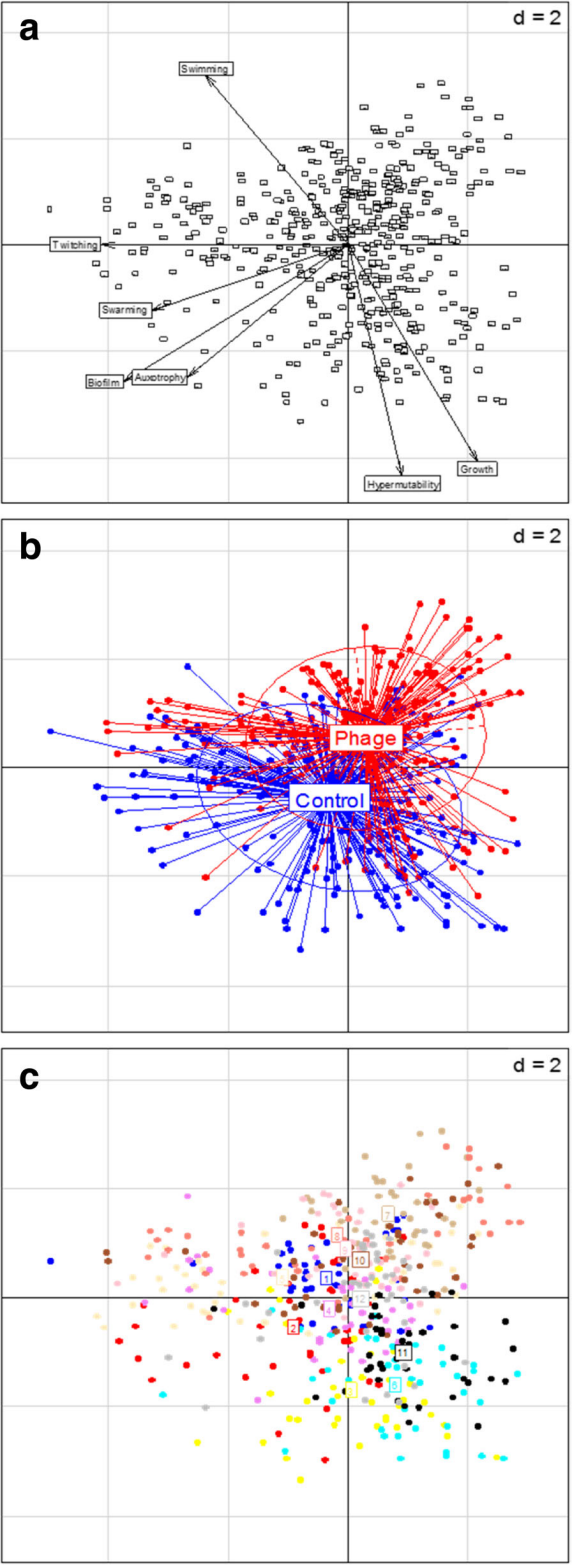
Exploratory principal component analysis of multivariate phenotype data. (**a**) Variable factor map, (**b**) individual factor map, labelled with experimental treatment and (**c**) individual factor map, labelled with population number. The Eigenvalues for the first and second components were 2.07 and 1.59, respectively

**Fig. 2:**
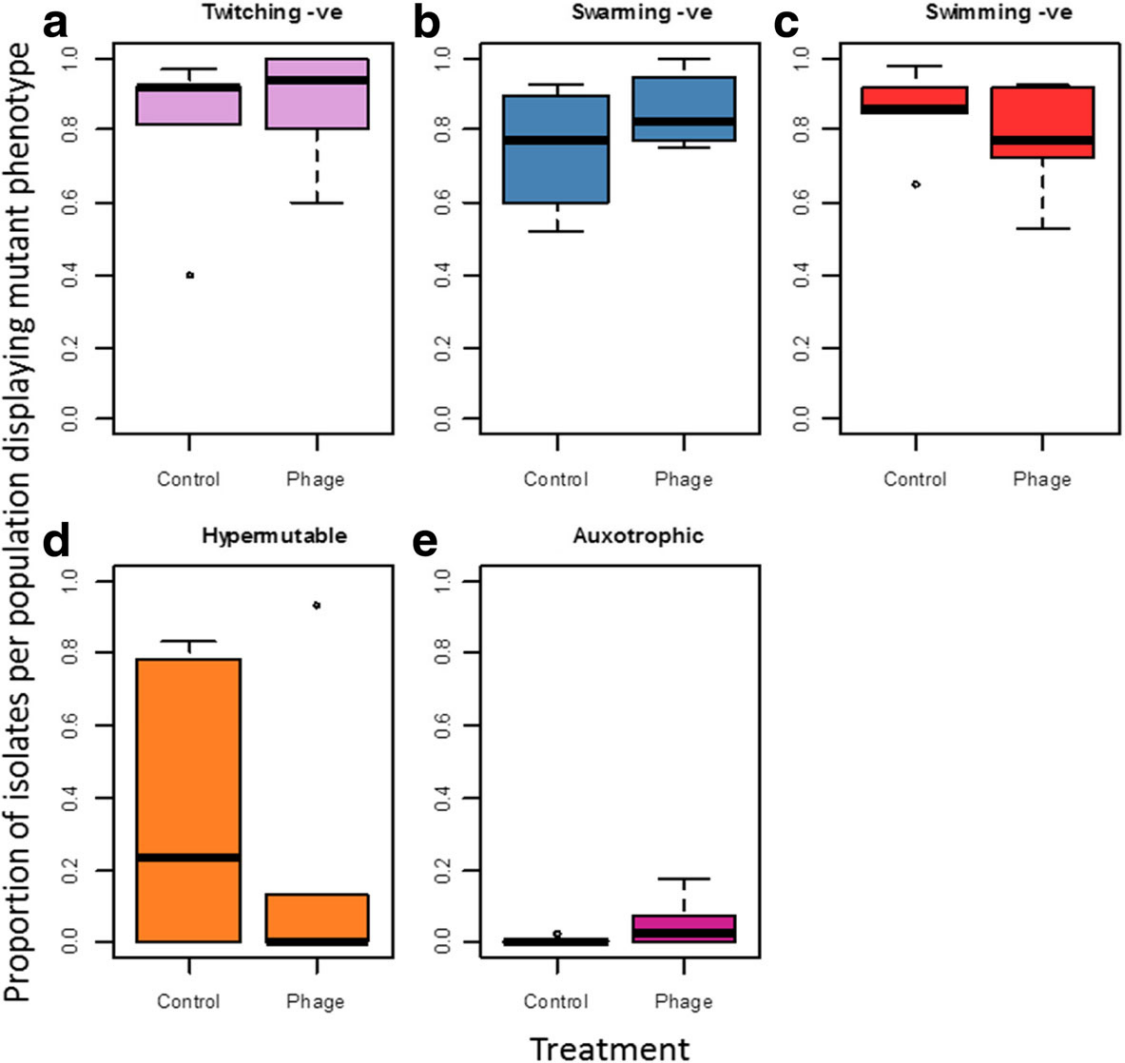
Boxplots of the proportion of isolates per population displaying the mutant phenotype for twitching motility (**a**), swarming motility (**b**), swimming motility (**c**), hypermutability (**d**) and auxotrophy (**e**). Thick black horizontal lines represent the median, the box represents the upper and lower quantiles, and the circles represent outliers. 40 isolates were tested per populations (6 populations per treatment)

In order to visualise variation between populations, a principal component analysis (PCA) was applied to the multivariate phenotypic dataset (Fig. 1). The first principal component was strongly associated with twitching motility (Fig. 1a). There was a large degree of overlap between isolates from the two treatments, suggesting that populations from the different treatments evolved along similar trajectories (Fig. 1b). While some degree of clustering by population was observed, extensive diversification within individual populations was evident (Fig. 1c). Many isolates exhibited loss of multiple phenotypes, but there was considerable variation in the proportion of mutants in each population (Fig. 2). However, there was no difference between the control and phage treatments for any of the phenotypes (Wilcoxon rank-sum test, *P* > 0.05).

**Fig. 3:**
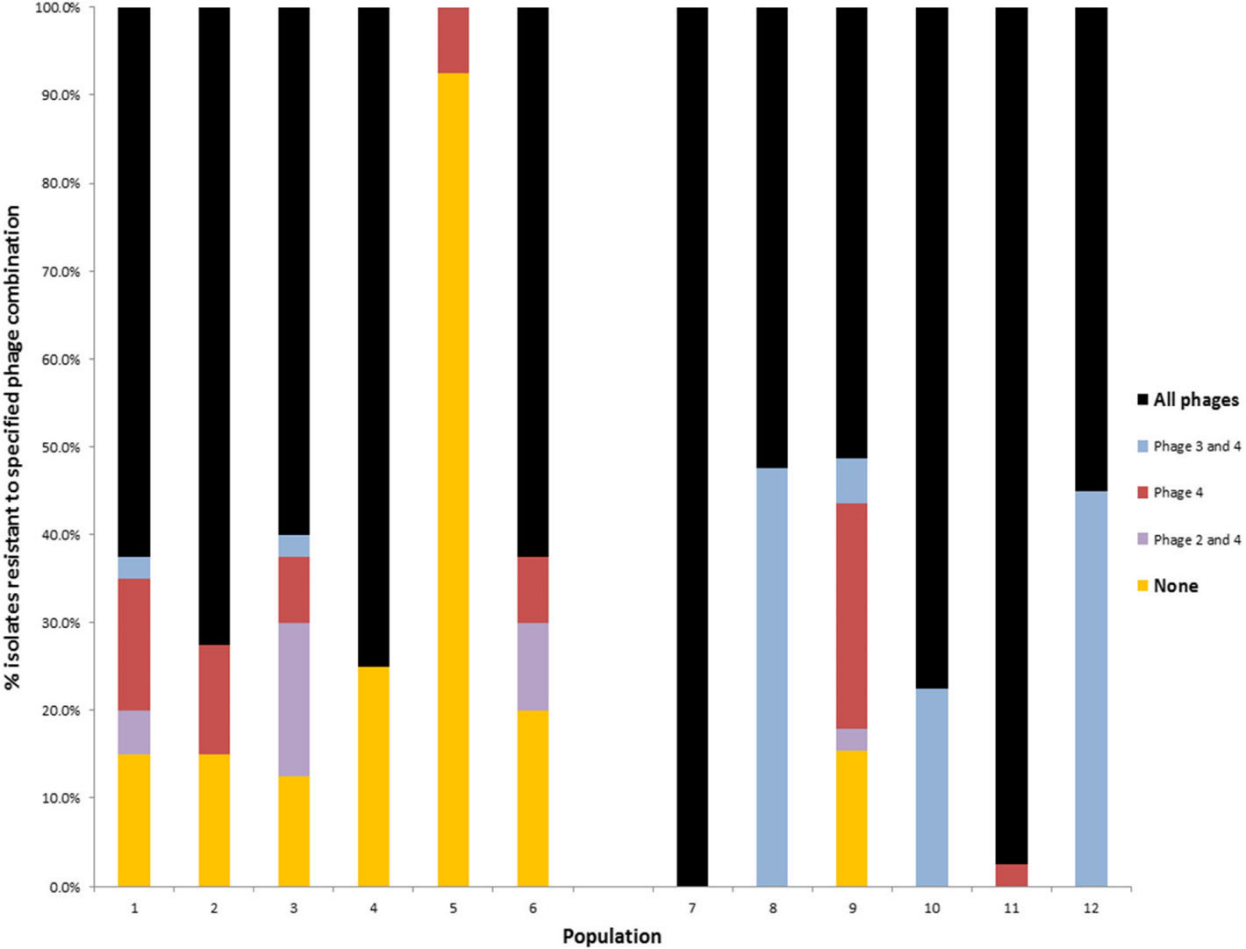
Phage resistance of endpoint isolates to LESφ2-4. Bars are coloured according to the particular phage combination to which resistance was observed. High titre pure phage stocks were spotted onto a soft-agar lawn of each isolate and scored as resistant if no lysis was observed after overnight incubation at 37 °C

Endpoint isolates were tested for resistance to each of the LES phages. Phage resistance was widespread in both treatments (Fig. 3). The majority of isolates were resistant to all three phages in all but one of the populations that was not exposed to phages (#5), in which 93% of isolates remained susceptible to all three phages.

### Resistance to antibiotics

**Fig. 4:**
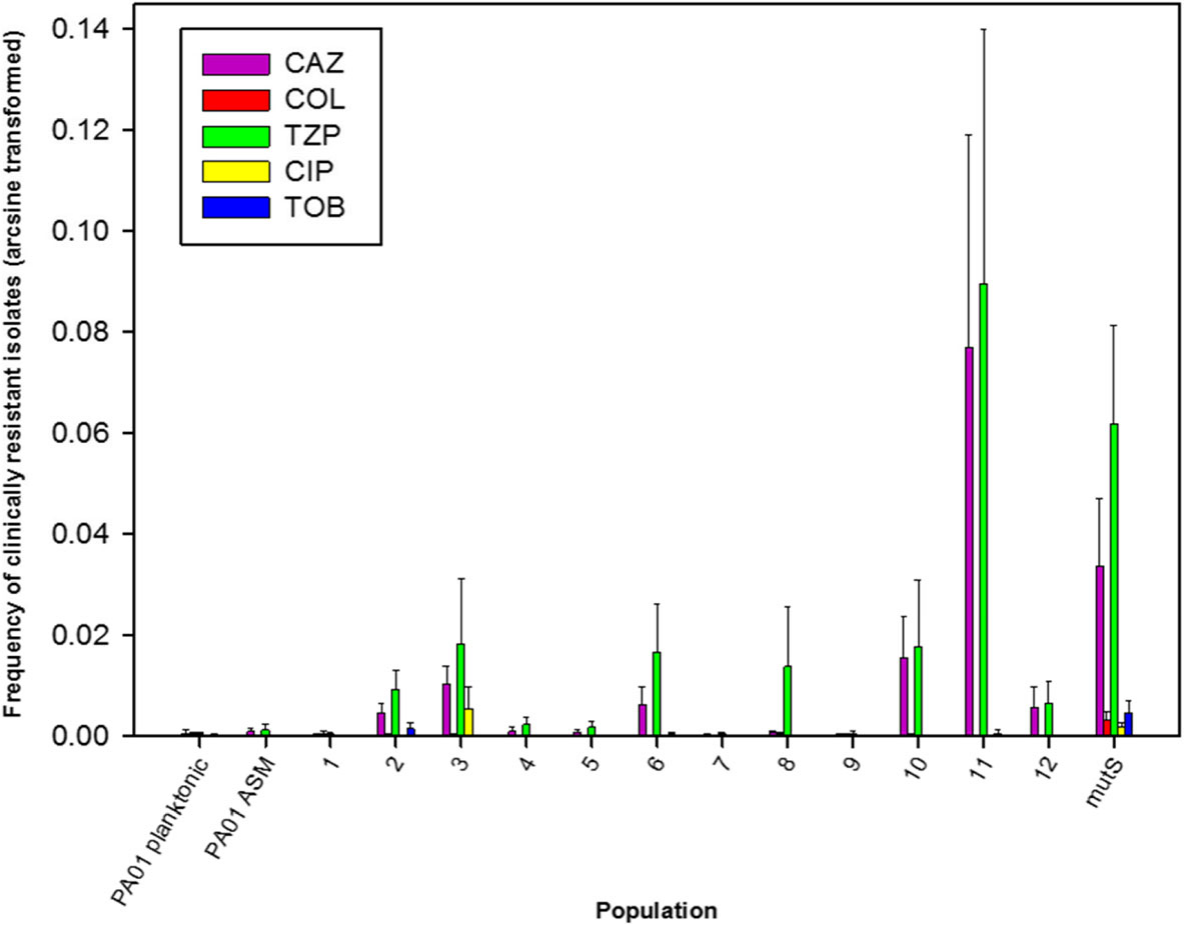
Frequency of bacterial cells in whole populations displaying clinical resistance to a panel of antibiotics (arcsine transformed). Frequencies were calculated after plating endpoint populations onto antibiotic free media, and media containing antibiotics at the clinical resistance breakpoint level. Planktonic PAO1 and PAO1 grown in ASM were included as negative controls. PAO1Δ*mutS* was included as a mutator strain control. Error bars ±1 S.E.M. Piperacillin/tazobactam (TZP); ceftazidime (CAZ); ciprofloxacin (CIP); tobramycin (TOB); colistin (COL)

Disk diffusion assays revealed that all of the isolates tested (40/40) from every population were as susceptible to a panel of antibiotics as the ancestor. The method was therefore adapted to look for resistant isolates that may be present at a low frequency in the population. Entire populations were spread on to agar plates containing antibiotics at clinically relevant resistance breakpoint levels. The frequency of antibiotic resistant isolates varied according to the population and the antibiotic (Fig. 4). The highest frequency of tazobactam-piperacillin resistance, observed in the phage-treated population #11 (0.08%), exceeded that seen with the control mutator strain, PAO1Δ*mutS*. Resistance to ceftazidime and tazobactam-piperacillin arose more frequently than resistance to other antibiotics. No meropenem resistant isolates were identified.

**Fig. 5:**
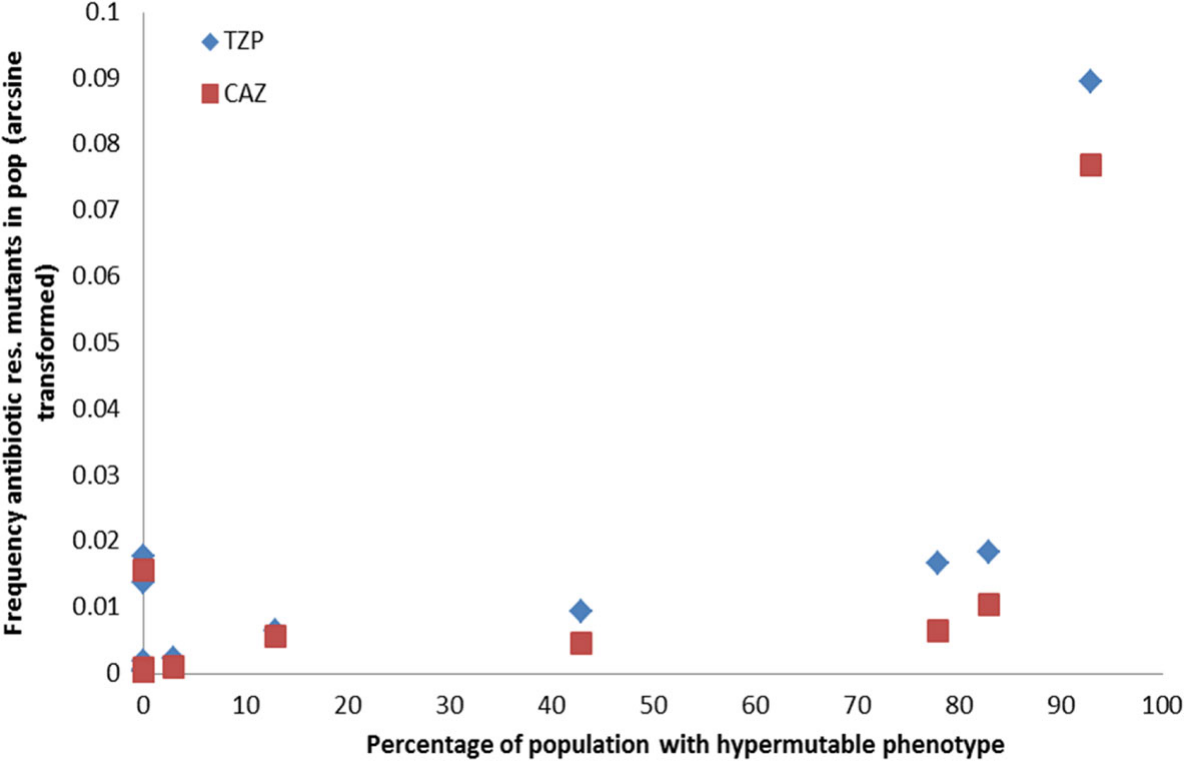
The correlation between the frequency of hypermutators in a population and the frequency of resistant isolates (arcsine transformed). Ceftazidime (CAZ); piperacillin/tazobactam (TZP); *n* = 12

**Fig. 6:**
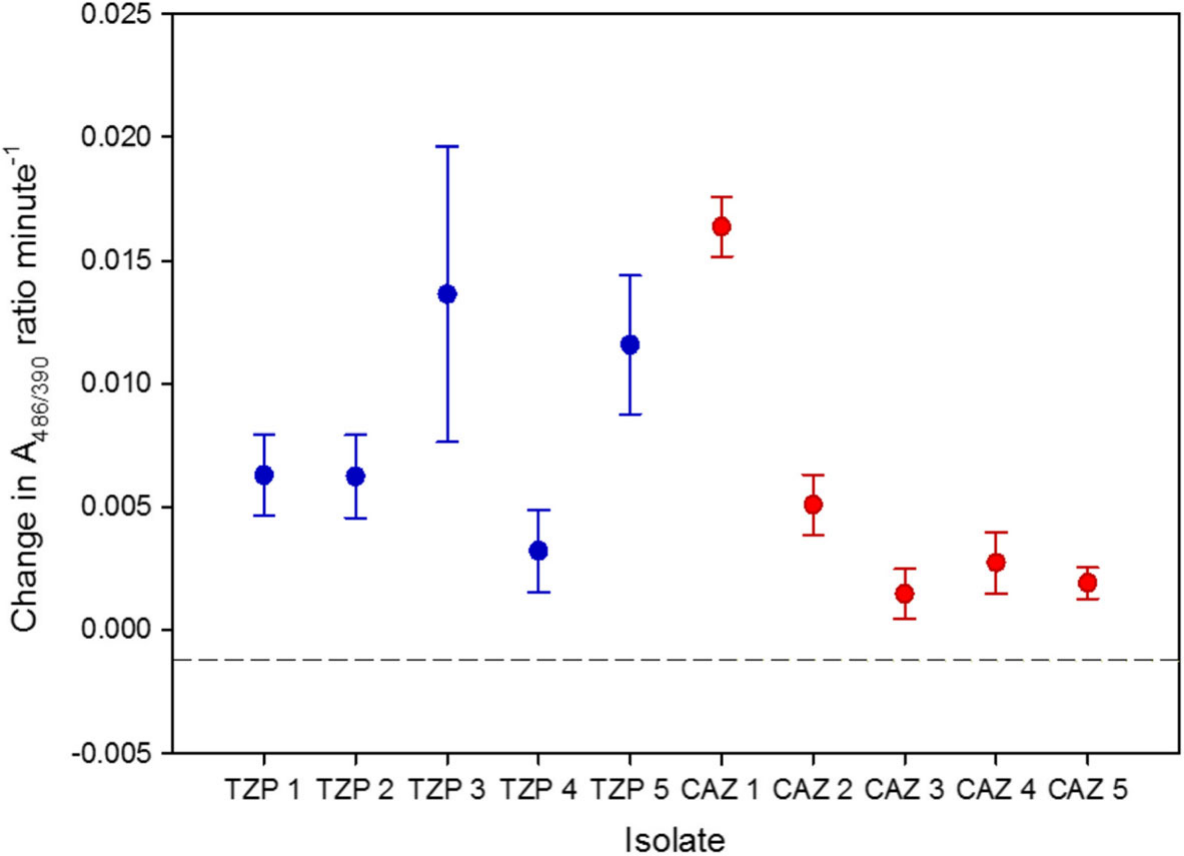
Beta-lactamase production in ceftazidime and tazobactam-piperacillin resistant isolates. Activity was measured using nitrocefin, in a chromogenic assay by calculating the change in A486/390 per minute. The dashed line indicates the value of the negative control (PAO1). Ceftazidime (CAZ); piperacillin/tazobactam (TZP). Bars indicate standard error

In order to assess the extent to which the mutator phenotype was linked to the high frequencies of antibiotic resistant cells, the frequency of hypermutators in a population was plotted against the frequency of isolates resistant to ceftazidime and tazobactam-piperacillin (Fig. 5). Higher frequencies of hypermutators were linked to frequencies of resistance against both tazobactam-piperacillin (Spearman’s rank correlation coefficient: *ρ* = 0.69, *p* < 0.05), and ceftazidime (Spearman’s rank correlation coefficient: *ρ* = 0.72, *p* < 0.01). To further characterise the nature of the spontaneously occurring antibiotic resistance, Minimum Inhibitory Concentration (MIC) values were determined for two sets of 20 colonies from population #11 identified as resistant to either ceftazidime or tazobactam-piperacillin. The MIC values for ceftazidime (32–128 mg l^−1^); and tazobactam-pipericillin (128–256 mg l^−1^) were at least two-fold higher than the clinical resistance breakpoints (8 mg l^−1^ and 16 mg l^−1^ respectively [32]). All isolates found to be resistant to one antibiotic were also resistant to the other. A chromogenic beta-lactamase detection assay was conducted on five randomly selected isolates from each antibiotic medium, using nitrocefin. Beta-lactamase production above the level of the control (PAO1) was observed in all of the isolates tested (Fig. 6). Antibiotic resistance data and data for beta-lactamase production are available in Additional File 2.

## Discussion

Consistent with clinical studies reporting extensive phenotypic diversity in *P. aeruginosa* populations recovered from the sputa of CF patients [10, 11, 13, 14], populations of *P. aeruginosa* grown in ASM exhibited substantial phenotypic heterogeneity. Notably we observed the evolution of many phenotypic variants that are also commonly found amongst isolates from CF patients. As with naturally occurring populations in the CF lung [10, 11], the majority of the diversity in our evolved populations was within populations rather than between populations. Although our previous genomic analyses suggested that the phages play a key role in accelerating the evolution of key phenotypes [26], our populations treated with or without phages evolved along similar trajectories, and converged upon similar suites of evolved phenotypes [26, 33].

Previous studies of CF isolates have reported loss of swimming, swarming and twitching motility [10]. Although it has been proposed that this loss of motility may be an immune avoidance strategy [4], the lack of any immune system in our model suggests that loss of motility may evolve, at least in part, because of other selective pressures. These could include the viscosity of the lung environment, though this remains a hypothesis yet to be tested. Although a role for type IV pili in biofilm formation has been suggested [34, 35], a study examining clinical isolates observed no correlation between biofilm production and motility [36]. Our observations suggest that type IV pili are not required for the formation of the kind of free-floating biofilms that occur in the ASM model.

A high frequency of hypermutators was observed in some evolved populations, but there were large differences between populations, ranging from 0 to > 90% hypermutators. An elevated mutation rate can be detrimental due to the increased risk of deleterious mutations, but hypermutability can reach fixation in a population by hitch-hiking with beneficial mutations [37], and can be advantageous in a novel environment, accelerating adaptation [38]. The mutator phenotype has been shown to occur during biofilm growth and may confer intrinsic fitness benefits, such as significantly enhanced microcolony growth [39], or overproduction of the H_2_O_2_-degrading enzyme catalase, leading to increased resistance to oxidative stress [40]. The hypermutability phentotype has also been linked with antibiotic resistance [41, 42], and in our study a clear correlation was observed between the frequency of hypermutators in a population and the frequency of clinically resistant isolates. Increased basal production of Beta-lactamase has been observed previously in mutator strains [43]. The finding that clinically resistant isolates are present at low frequency in hypermutable populations, even in the absence of antibiotic selection, suggests that factors other than antibiotic therapy per se can drive antimicrobial resistance in chronic lung infections. The role of mutator phenotypes in driving evolution in *P. aeruginosa* surface-attached biofilm systems, including an increase in the frequency of mutations implicated in antibiotic resistance, has been described previously [39]. Evolution of antibiotic resistance has also been shown during biofilm growth for *Escherichia coli* [44]. Here, we demonstrate the development of resistance in a free-floating biofilm model systems designed to mimic sputum.

The diversification observed in ASM over this relatively short timescale could be driven by growth in a biofilm. It is known that spatially structured environments, like the viscous ASM, can select for the evolution of diversity [45]. Previous studies further suggest that *P. aeruginosa* diversifies when grown as a surface-attached biofilm, and this diversity helps protect against oxidative stress [24]. It has been suggested that this was due to the “insurance effect”, which posits that biodiversity has a buffering effect over time on productivity, as well as raising the overall mean productivity of an ecosystem [46]. However, the applicability of the insurance hypothesis to bacterial biofilms has been questioned [47]. Our observations suggest that diversification of biofilm populations, especially those that have evolved hypermutability, could potentially increase their evolutionary responsiveness in the event of antibiotic treatment by providing standing genetic variation for antibiotic resistance upon which natural selection can act.

## Conclusions

We have demonstrated that many of the phenotypic adaptations observed amongst *P. aeruginosa* isolates from CF chronic infections occur during evolution in a model designed to mimic the CF sputum environment, even in the absence of any selection due to host responses or antibiotics. Although we have shown previously that phages can alter the mode and rate of adaptive evolution [26], comparable sets of phenotypes eventually occurred both in the presence and absence of phages. It is, however, worth noting that similar phenotypes can be driven by sub-inhibitory concentrations of antibiotics in non-biofilm systems [48]. In CF, it has been proposed that the maintenance of phenotypically diverse *P. aeruginosa* populations may be a consequence of the spatial heterogeneity of the lung, leading to regional isolation of separately evolving communities [15]. Here we show that similar population heterogeneity occurs in an artificial sputum biofilm system, emphasizing the potential utility of this model as a tool to facilitate a better understanding of the factors driving *P. aeruginosa* adaptation and diversification during infections.

## Methods

### Bacterial strains, bacteriophages and growth conditions

The laboratory reference strain *P. aeruginosa* PAO1, originally isolated from a wound in Melbourne, Australia in 1954 [49], was used as a model host because it is known to be fully susceptible to LES φ2-4. *P. aeruginosa* LESB58 was isolated from a CF patient in Liverpool in 1988 [19]. Bacteriophages were isolated from the *P. aeruginosa* LESB58 as described previously [50]. The challenge experiment was performed using PAO1 cultures grown in ASM [22, 30] at 37 °C, 60 rpm for 96 h periods as described previously [26]. Prior to the challenge experiment and in follow up experiments, all bacterial strains and phages were grown and propagated in standard Luria Bertani broth (LB). Phage suspensions (1 × 10^8^ – 1 × 10^11^ p.f.u ml^−1^) were stored in LB at 4 °C.

### Challenge experiment

Twelve replicate ASM cultures (hereafter referred to as populations) were initiated by inoculation of approximately 5 × 10^7^ cells of mid-exponential phase PAO1 into 5 ml ASM, followed by 24 h incubation (37 °C, 60 rpm). Equal numbers of each LES phage (LES φ2 – 4) were then added, once only, to 6 replicates (populations 7–12), to a total multiplicity of infection (MOI) of 0.1. The remaining six replicates were designated phage-free controls (populations 1–6). Each population was incubated for a further 72 h before degradation of biofilm structures using Sputasol (Oxoid) as described previously [30]. The biofilm homogenate was transferred (1:100) into fresh ASM (12 × 5 ml) and incubated for a further 96 h. This process was repeated every 96 h for 120 days (30 transfers and approximately 240 bacterial generations).

### Phenotypic diversity of endpoint populations

At the end of the challenge experiment, 40 evolved isolates were obtained from each population by plating onto Columbia media and patching of randomly selected colonies onto fresh Columbia media. All isolates were stored in LB with 30% (v/v) glycerol at −80 °C. A total of 240 isolates for each treatment were subjected to a series of phenotypic tests.

For growth rate in LB, overnight cultures were diluted 1:100 and 200 μl added to wells (*n* = 4 per isolate) of a clear-bottomed 96 well plate. The plate was held at 37 °C, with shaking at 100 r.p.m. in an Omega fluostar plate reader. The OD600 was measured every 5 min and the doubling time in the exponential phase of growth calculated for each well. Two biological replicates were performed per isolate. Ten isolates were tested per population.

For auxotrophy, a single colony was patched onto M9 minimal media and incubated at 37 °C. Isolates displaying no growth after 18 h were classed as auxotrophic. For hypermutability, 10-fold serially diluted overnight cultures grown in LB were spotted onto LB agar plates containing 300 μg ml^−1^ rifampicin. Plates were incubated overnight at 37 °C. As described previously [12], isolates were classed as hypermutable if growth on rifampicin was comparable to PAOΔ*mutS* (ie. different by less than 10-fold), a known hypermutator.

For swarming motility, a small portion of a colony was lightly touched on to the surface of media containing 0.5% (w/v) bacteriological agar as described previously [51]. Plates were incubated for 16 h at 37 °C and the diameter of the swarm was measured at the widest point, using a strain PAO1 *pilA* mutant as a negative control. An isolate with a diameter <6 mm was considered swarming deficient. For swimming motility, a small portion of a colony was lightly touched to the surface of a swimming plate [52]. Plates were wrapped loosely in clingfilm to prevent dehydration, and incubated without inversion for 14 h. The visible diameter of bacterial growth was measured at the widest point. Using the non-motile *P. aeruginosa* LESB58 was used as a negative control, an isolate with a diameter <20 mm was considered swimming deficient. Twitching motility was measured in duplicate as described previously [53], using ancestral PAO1 as a positive control. Isolates with a zone diameter <10 mm were considered to have impaired twitching motility.

To test bacterial resistance to the phages used in this study (LESφ2, φ3 and φ4), a modified version of the phage spot assay was used. 20 μl of 10-fold diluted overnight culture was mixed with 200 μl agar overlay and poured into a well containing 1 ml bottom agar, in a 25 well square petri dish (Fisher Scientific). High titre (>1x10^10^ pf.u. ml-1) preparations of each phage were spotted on top. Isolates were classed as resistant to a phage if there was no evidence of bacterial lysis after overnight incubation at 37 °C.

### Antibiotic susceptibilities

Susceptibilities to six antibiotics were determined using the disc diffusion method, as described previously [11]. The antibiotics were: piperacillin/tazobactam, ceftazidime, meropenem, ciprofloxacin, tobramycin and colistin. To detect resistant isolates at a low abundance in a population, a modified version of the agar dilution method was developed. Whole population cultures were adjusted to the turbidity equivalence of a McFarland standard 0.5 (Beckton Dickinson), and 100 μl was plated directly onto Mueller-Hinton Agar plates containing antibiotics at the breakpoint levels for clinical resistance [32]. After overnight growth at 37 °C, colonies growing on antibiotic-containing media were calculated as a proportion of total population counts. “Planktonic” PAO1 grown in LB and PAO1 grown in ASM were included as controls.

### Beta-lactamase assay of beta-lactam resistant isolates

Isolates were passaged twice through antibiotic-free media (LB agar) to ensure basal (as opposed to induced) beta-lactamase levels were being studied. Cultures were grown in LB to an Absorbance at 600 nm of 0.3, and nitrocefin (Merck-Millipore) was added to a final concentration of 51.6 μg ml^−1^. As the λmax of nitrocefin changes from 390 nm (in the absence of beta-lactamase) to 486 nm upon hydrolysis, the absorbance at both wavelengths was measured every 5 min, for 35 min. The A486 nm/390 nm ratio was calculated for every time point as an indicator of beta-lactamase production. Five biological replicates were performed for each isolate.

### Analysis of molecular variance (AMOVA)

At the end of the challenge experiment, 40 evolved isolates were selected at random from each population and screened for eight phenotypic traits (the three types of motility, auxotrophy, hypermutability, resistance to each of the three phages). Isolates were classified as different haplotypes based on the combination of phenotypic traits displayed. For each trait, isolates were scored a 0 if they displayed the same phenotype as the ancestor, or a 1 if they displayed a mutant phenotype. Here we define a haplotype as a unique combination of phenotypic traits. The evolutionary distance between the haplotypes was estimated by creation of a Euclidean distance matrix. AMOVA was performed using the ade4 package in R [54]; the distance matrix was partitioned into sub-matrices for the various subdivisions of the data, including treatment (control or phage-treated), population and individual isolates. The sums of squares were computed and analysed in a nested ANOVA framework and significance tested by Monte-Carlo permutation tests (49 permutations). Such an approach has been used previously to assess diversity of clinical *P. aeruginosa* isolates, using phenotype data [11].

### Principal component analysis (PCA)

Phenotypic data from the host-phage coevolution experiment resulted in large, multivariate datasets. To explore the underlying structure of the data and elucidate the factors that are responsible for much of the variance, PCA was used as an exploratory data analysis tool, using the ade4 package [54] in R. Complete datasets (i.e. data for all 40 isolates within each population) were available for all but two of the variables (biofilm formation and doubling time in LB). For the incomplete variables, only the first 10 isolates of every population were tested, due to the large amount of time and work required to test these specific phenotypes. PCA in R cannot proceed with missing values, but numerous methods exist to allow missing values to be ignored or replaced. Multiple imputation was chosen as a method to replace the missing data, using the mi package [55] in R, as it enables existing data to be retained. Data were plotted with dudi PCA scatter plots, and the independent variables (phage-treatment and population) overlaid, using the s.class function. Data for seven phenotypes were included in the PCA analysis: swimming, swarming and twitching motility, hypermutability, growth rate, biofilm formation and auxotrophy.

## Additional files

Additional file 1: Phenotypic data from this study. (XLSX 139 kb)
Additional file 2: Antimicrbial susceptibility data and data for beta-lactamase production. (XLSX 18 kb)

## Abbreviations

AMOVA: Analysis of molecular variance
ANOVA: Analysis of variance
ASM: Artificial sputum medium
CF: Cystic fibrosis
LB: Luria Bertani
LES: Liverpool Epidemic Strain
PCA: Principal component analysis
